# Crowdsensing in Smart Cities: Overview, Platforms, and Environment Sensing Issues

**DOI:** 10.3390/s18020460

**Published:** 2018-02-04

**Authors:** Oscar Alvear, Carlos T. Calafate, Juan-Carlos Cano, Pietro Manzoni

**Affiliations:** 1 Department of Computer Engineering, Universitat Politecnica de Valencia, 46022 Valencia, Spain; calafate@disca.upv.es (C.T.C.); jucano@disca.upv.es (J.-C.C.); pmanzoni@disca.upv.es (P.M.); 2 Department of Electrical Engineering, Electronics and Telecommunications, Universidad de Cuenca, Cuenca 010150, Ecuador

**Keywords:** Smart city, Internet of Things, crowdsensing, sensor design

## Abstract

Evidence shows that Smart Cities are starting to materialise in our lives through the gradual introduction of the Internet of Things (IoT) paradigm. In this scope, crowdsensing emerges as a powerful solution to address environmental monitoring, allowing to control air pollution levels in crowded urban areas in a distributed, collaborative, inexpensive and accurate manner. However, even though technology is already available, such environmental sensing devices have not yet reached consumers. In this paper, we present an analysis of candidate technologies for crowdsensing architectures, along with the requirements for empowering users with air monitoring capabilities. Specifically, we start by providing an overview of the most relevant IoT architectures and protocols. Then, we present the general design of an off-the-shelf mobile environmental sensor able to cope with air quality monitoring requirements; we explore different hardware options to develop the desired sensing unit using readily available devices, discussing the main technical issues associated with each option, thereby opening new opportunities in terms of environmental monitoring programs.

## 1. Introduction

Smart cities are revolutionising our view of the world, and their functioning achieves a very high level of integration, coordination, and cooperation between ordinary objects, providing them with some degree of intelligence. This novel paradigm provides a plethora of systems and technological tools aimed at increasing our life quality, minimising the environmental impact of everyday activities, and optimising resource usage. Such effects are more noticeable in urban areas with millions of citizens, the so called *mega cities*, which in the near future will be more and more common [1]. The main concept behind a Smart City is the integration of the physical world with the virtual world [2]. This is achieved by providing additional capabilities such as environmental sensing and automatic behaviour to common objects, allowing to capture and to analyse the data from the real world to ensure a better operation of the virtual one.

**Figure 1:**
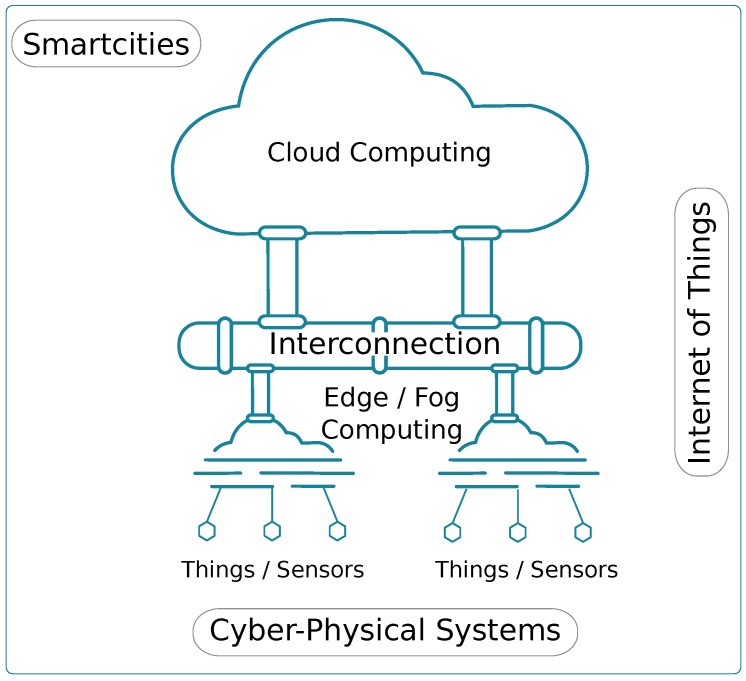
Smart city structure.

There are several technologies/perspectives that simplify the process of creating a Smart City. Figure 1 provides a global overview of available technologies from diverse perspectives, which cover different aspects that allow creating a Smart City. Thus, in a Smart City, all daily objects, called things, are equipped with extra capabilities, usually sensing and/or acting capabilities, along with communication capabilities, to share information and to optimise their functional operation. This way, and from a communications perspective, the Internet of Things (IoT) [3] focuses on the intercommunication between all things, as well as on the communication between things and data servers (Cloud or Fog/Edge). On the other hand, from an operational perspective, Cyber Physical Systems (CPS) focus on the integration of these physical things with the computational process [4,5,6] to improve its functionally. Finally, from a service perspective, Cloud Computing [7,8] and Edge/Fog [9,10,11] Computing focus on the data processing, and on the structure of the Central servers or Local devices. In the remainder of the paper, we will focus on the Internet of Things perspective, analysing available technologies and their adequacy in terms of implementation.

Any city has several areas of concern to the authorities. In a smart city, all of these areas must have some level of intelligence to minimise management efforts. Thus, there are various subareas of interest including Smart Governance, Smart Mobility, Smart Utilities, Smart Buildings, and Smart Environment, where the adoption of this paradigm can have a clear impact, being highly beneficial [12,13].

Sensing processes are one of the most important tasks in a smart city because they allow retrieving the different parameters involved in different control processes. Examples of such processes include transportation, energy management, air conditioning, etc. However, controlling air pollution in smart cities stands out as a key issue, as it has severe consequences on human health, thereby making environment sensing a critical task and a prominent service.

Currently, controlling pollution levels is an on-going effort undertaken by most European cities, which invest a considerable amount of money in controlling the different hazards produced by poor air quality. Considering these aspects, an index known as AQI (Air Quality Index [14]) was created to classify the air quality, and it specifies different healthy risks, from Low to Very High.

Air quality assessment mainly relies on static monitoring stations, meaning that most cities are endowed with at least one of these stations. Overall, there are about 1500 air quality monitoring stations in Europe. However, the installation and maintenance of these stations is quite expensive. In addition, installing new air monitoring stations is tough since, in crowded places, there is no room for allocating them, meaning that they are typically installed in remote locations, such as parks or sparsely populated areas, which is prone to produce data with little representativeness for the overall population.

There are several air pollutant types, and various techniques can be adopted to measure them. In general, air pollutants can be of two types: (i) primary air pollutants, which are gases or particles emitted directly into the atmosphere, including carbon monoxide (CO), carbon dioxide (CO${}_{2}$), particulate matter smaller than 10 microns (PM${}_{10}$), or particulate matter smaller than 2.5 microns (PM${}_{2.5}$); and (ii) secondary air pollutants, which are gases produced by a chemical reaction between primary pollutants and some environment element, including ozone (O${}_{3}$), which is produced by the combination of nitrogen oxides (NOx), Oxygen (O${}_{2}$), Volatile Organic Compounds (VOC), and sunlight [15]. In addition, some pollutants are gases (i.e., CO${}_{2}$, CO, and Ozone), while others are suspended particles in the air (PM${}_{10}$, and PM${}_{2.5}$). Usually, the process of gas monitoring relies on chemical elements that react to the presence of these gases, while the monitoring of suspended particles mostly relies on optical detection procedures.

By embracing the Smart City paradigm, crowdsensing becomes a solution able to cope with air pollution monitoring since this novel paradigm assumes that a significant number of users perform collaborative sensing tasks, thereby collecting data from different populated locations while doing their daily activities. The collected data is periodically transmitted to a central server (Cloud) for data storage and processing. Overall, this strategy implies that the sensors used must be cheap and tiny enough for comfortable and easy transportation. Otherwise, it becomes hard to achieve a widespread distribution and adoption. Besides, there must be a communications link for transmitting the acquired data to a cloud-based server, where data are constantly being stored and processed.

To fulfil the first two requirements concerning price and size, we get advantage by using emerging prototyping platforms such as Raspberry Pi or Arduino, which allow achieving the aforementioned goals when combined with low-cost sensors available on the market. Such solutions, besides being inexpensive, have the advantage of using compact, powerful, and easy-to-use hardware that is widely adopted nowadays both in research and industry. These compact platforms can also be integrated in vehicles, i.e., cars, bikes, or public transportation, making it easy to monitor different air pollutants while the vehicle travels around the city.

On the other hand, smartphones are nowadays widely adopted devices that have become ubiquitous. Thus, it becomes interesting to exploit their communication interfaces, such as Wi-Fi or Cellular, for transmitting the acquired data from mobile sensors. With these issues in mind, mobiles sensors must be able to create a communication link with smartphones to make such interaction possible.

Recent literature suggests that, although there are several hardware options for creating different types of sensors, there are none specific for air pollution monitoring from a crowdsensing perspective. Hence, it becomes necessary to analyse the different options for creating a small, low-cost, mobile sensor able to communicate with off-the-shelf smartphones in an Internet of Things type of environment.

This paper is organised as follows: Section 2 presents a literature overview of crowdsensing projects. Section 3 provides an overview of the current IoT platforms and protocols that are able to support environment-sensing solutions under the crowdsensing paradigm. Section 4 presents an analysis of available architectures and technologies for creating a crowdsensing system while defining the basic requirements for it. Section 5 presents a comparison of possible hardware configurations for creating a mobile sensor able to perform air monitoring, transmitting the collected data via some mobile sink (e.g., a smartphone) towards a datacenter for storage and processing. Section 6 studies the main requirements of this type of sensors, thoroughly assessing some developed configurations that fulfil the aforementioned requirements. Finally, Section 7 presents the conclusions of the present work.

## 2. Crowdsensing Architectures in the IoT Context: Literature Overview

Following the Smart City paradigm, and focusing on the data collection domain, the concept of crowdsourcing has been introduced to refer to scenarios where a large group of people, through different devices and technologies, actively participate in the data acquisition process [16]. Once data are collected, they are sent to a central server for analysis, and feedback will eventually be returned to citizens through actions and services aimed at improving their life quality.

Crowdsensing is a subtype of crowdsourcing where sensors are the actual sources of the data gathered [17]. If air quality sensors are used, crowdsensing becomes a good alternative to traditional stationary air quality stations whereby small sensors are distributed to a large group of people that seamlessly contribute to the system while doing their everyday tasks [18].

In the literature, we can find several works related to crowdsensing systems applied to air monitoring. Air-cloud [19] is a system to monitor the concentrations of PM${}_{2.5}$ using crowdsensing in China. It focuses on the analysis of the mechanical sensor design to optimise air reception, as well as on data fusion techniques. The sensor calibration process relied on neural network tuning using laboratory measurements as input.

U-Air [20] shows how to analyse the data obtained from different sources, such as traffic levels, weather conditions, and pollution using various Big Data techniques, evidencing how these techniques allow inferring environmental pollution levels with better granularity.

Researchers from Zurich University [21] have also designed a prototype to monitor ozone levels using a sensor that is connected to the smartphone via an USB cable. The Smart City project in Serbia [22] seeks similar goals, relying on a commercial sensor by Libelium [23] for measuring various air pollutants using the public transport system.

Devarakonda et al. [24] propose a system with two types of sensors: (i) a sensor based on Arduino Mega128 to install in vehicles that costs about $700; and (ii) a personal sensor that is smaller than previous ones, and that costs about $400 for end-user applications. In this work, Google technology is used for data processing.

Mead et al. [25] analyse the behaviour of electrochemical sensors to monitor air pollution in urban scenarios. They design two types of sensors (static and mobile) based on the PIC18F67J10 microchip, and they show how to deal with electrochemical sensors.

Manna et al. [26] propose a sensor to monitor air pollution on roads, and to track vehicles which cause pollution, by using a sensor based on Arduino, an electrochemical CO gas sensor, and RFID technology.

In addition, we can find several technological approaches for fabricating small sensors. For instance, Hjiri et al. [27] use Al-doped ZnO (AZO) nanoparticles to create a highly sensitive CO gas sensor. Instead, Borini et al. [28] use graphene oxide to create humidity and temperature sensors with ultrafast response times (30 ms). Chen et al. [29] analyse the use of nanowires to fabricate gas sensors due to their characteristics: ultrasensitive, higher selectivity, low power consumption, and fast response. In addition, Zaatar et al. [30] show how to use the fiber optic evanescent wave to monitor air pollution.

Even though there are different studies that provide a wide set of approaches for air pollution monitoring through crowdsensing, or that provide an isolate analysis of sensing technologies, there is no specific hardware solution that is widely available for regular users, and different alternatives have not yet been compared in a detailed manner. Thereby, an exhaustive analysis of the different solutions available to create a small, low-cost device endowed with both air quality sensors and communication capabilities, would represent a step forward in this challenging and fascinating area.

## 3. Internet of Things Protocols Overview

In recent years, the Internet of Things (IoT) has become one of the most challenging research topics, offering a wide range of novel solutions for Smart Cities [3,31]. These proposals mainly analyse the intercommunication between devices, and involve a large variety of domains like home-based solutions [32], intelligent transportation systems [33], healthcare [34], safety and security [35,36], industrial control [37], and environmental monitoring. Thus, the analysis of the sensor design must be able to cope with IoT protocols, which will be described in this section.

The main principle underlying the IoT paradigm is that all “things” are, or must be, connected to the Internet, and interact with each other to create developed areas that promote sustainability and a high life quality in multiple key areas [13,38].

**Figure 2:**
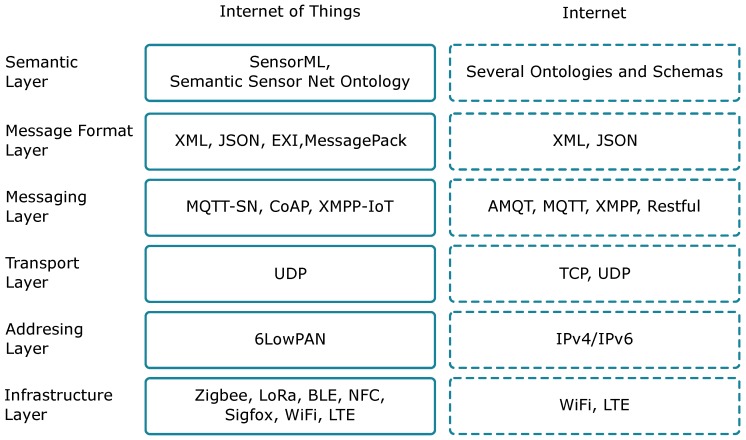
IoT protocols.

The main characteristic of these things is that they are constrained devices such as small sensors, meaning they have restricted processing/storage capacity, restricted battery, restricted communication characteristics, i.e., Low Bandwidth, Low Data Rate, Low Coverage, etc. With this in mind, the Internet of Things has created a subset of protocols divided into various layers, similar to the traditional Internet stack, but taking into account the restrictions of the Internet of Things in terms of processing, battery capacity, and communication capabilities of embedded devices. Figure 2 summarises the different layers defined for IoT, and the differences towards the traditional Internet: (i) the Infrastructure Layer typically relies on wireless technologies, like ZigBee, LoRa, or Bluetooth Low Energy (BLE); (ii) the Addressing Layer focuses on the analysis of the addressing issues to achieve compatibility with Internet protocols; (iii) the Transport Layer is the same than for the TCP/IP (Internet) protocol stack, so either TCP or UDP are available, although UDP is typically used; (iv) the Messaging Layer defines protocols to transmit data towards the servers; (v) the Message Format Layer defines encoding types to store and transmit data; and (vi) the Semantic Layer defines the structure of the data.

Below, we will describe the most important protocols involved in the Internet of Things at these different layers.

### 3.1. Infrastructure Layer

Currently, there are several communication technologies for the Internet of Things. Notice that, while any communications technology would allow us to create a network for IoT, not all of them take device restrictions into account. Theoretically, 5G allows a lot of possibilities to be offered in the context of the Internet of Things [39]. Similarly, new technologies such as LoRA or SIGFOX allow network sensors to remain connected due to their large coverage.

Below, we make a brief analysis of the possible wireless technologies for IoT.

**5G Network** [40] is the fifth generation cellular network architecture, designed to support great amounts of data, high speed, configurability, etc. from new emerging technologies such as Internet of Things. Currently, it is in the first phase, where new standards and services will be defined, but soon it shall become the default cellular network technology.

**ZigBee** [41] is based on the **IEEE 802.15.4** standard, and it was designed for Wireless Sensor Networks. Its main characteristics are its small size and low power consumption. Usually, the transmission range can vary from 10 to 100 m, depending on the output power. The main drawback of this technology, though, is that current smartphones are not equipped with ZigBee interfaces.

**Wi-Fi** [42] is based on the IEEE 802.11 standard, and it was designed for Wireless Local Area Networks. The evolution of this technology provides several variants operating in the 2.4 GHz or in the 5 GHz band, being currently the 802.11n version the most widespread option. The transmission range for standard interfaces is about 100 m.

**LoRa** [43] is a LPWAN (Low-Power Wide-Area Network) technology designed to optimise different aspects such as communication range, battery lifetime, and costs, supporting thousands of devices headed for the Internet of Things in several domains including sensing, metering, and machine-to-machine (M2M) communications.

Theoretically, LoRa achieves a transmission range of more than 15 km in rural environments, and of more than 2 km in dense urban areas. Its bandwidth ranges between 250 bps and 50 Kbps in different frequencies: 169 MHz, 433 MHz, and 868 MHz in Europe, and 915 MHz in North America.

**SIGFOX** [44] is an emerging technology that offers a proprietary telecommunications network to support Internet of Things solutions. It was designed for LPWA (Low-Power Wide-Area) networks operating in the ISM 868 MHz band, reaching distances greater than 1 km. Since the selected ISM band is restricted, the communications could be of up to 12 bytes per message, and up to 140 messages per day.

**Near Field Communications (NFC)** [45] was designed for communications between two nearby devices (closer than 4 cm). It main target applications are smartphone-based payments and IoT solutions such as access control, or inventory systems. However, its distance requirements and intermittent connectivity features make it a poor option for our purposes.

**Bluetooth** [46] was designed for Personal Area Networks, purposely having a maximum coverage range of 10 meters by default. Currently, it is used for transmitting information between personal devices, such as smartphones, smartwatches, and headsets.

**Bluetooth Low Energy** [47], or **Bluetooth Smart**, is the name under which Bluetooth version 4 is known. Its main advantage, when compared to previous versions, is using very low power, being nowadays one of the best options for IoT applications. Similarly to previous Bluetooth specifications, the coverage range is of 10 m.

### 3.2. Addressing Layer

The addressing layer defines the logic address of the packets by assigning a specific address to all possible nodes. These protocols deal with the packet forwarding problem, which in the TCP/IP model is handled by IPv4 and/or IPv6.

**6lowPAN** [48] (IPv6 over Low power Wireless Personal Area Networks) allows using IPv6 over networks based mainly in the IEEE 802.15.4 standard.

It is designed for resource-constrained devices by reducing the size of the address to 64 or 16 bits, depending on whether it is for a Local Network or a Personal Area Network, respectively; it uses default values for specifying the network.

### 3.3. Messaging Layer

The messaging layer defines protocols for data transmission systems considering IoT restrictions.

**RESTful** [49] (Representational state transfer) is a Web-based architecture to exchange or to manipulate Web resources through a textual representation using preset stateless operations; it means that each message must have all the required information to complete the request. It follows a client/server model based on the HTTP protocol, and relies on its functions: (i) GET, to retrieve a resource; (ii) POST, to create a resource; (iii) PUT, to change the state of a resource; and (iv) DELETE, to delete a resource. The data representation typically adopts the XML or JSON formats.

**Figure 3:**
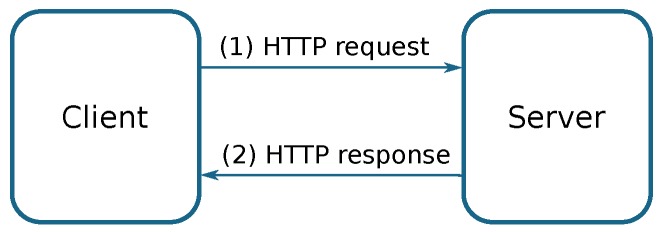
Basic RESTful architecture.

Figure 3 presents a basic overview of a Restful Architecture under the client/server model.

There is a specification [50] for constrained nodes and networks called Constrained RESTful Environments (CoRE) Link Format. It specifies a set of links to discover resources, and to access these resources in a Machine-to-Machine (M2M) environment.

**MQTT** [51] (Message Queue Telemetry Transport) is a lightweight messaging protocol based on the publisher/subscriber scheme that runs on top of the TCP/IP protocol. It is also designed for constrained networks with limited bandwidth.

The MQTT is composed by three elements: publishers, subscribers, and a message broker. A subscriber, which wants to receive a message related to a specific topic, must subscribe to the message broker; next, when a publisher sends/publishes a message related a certain topic, it is transmitted to all subscribers subscribed to this topic.

MQTT has three transmission Quality of Service (QoS) levels: (i) QoS 0: At most once. The message is sent once, but it does not check for ACKs to confirm message reception. (ii) QoS 1: At least once. The message could be sent more than once to each subscriber. (iii) QoS 2: Exactly once. The message is sent exactly once using four-way handshaking.

**Figure 4:**
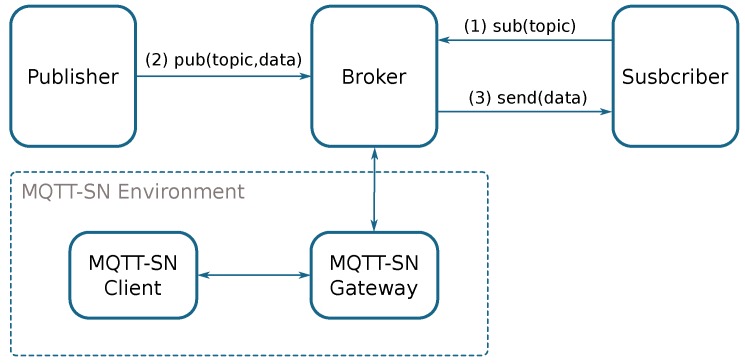
Basic MQTT (Message Queue Telemetry Transport) architecture.

MQTT-SN [52] (MQTT Sensor Network) has been designed to be similar to MQTT, but considering the restrictions of wireless communication environments, such as limited bandwidth, short message length, etc., running over UDP or on Non-IP environments. For interoperating with standard MQTT environments, it needs an MQTT-SN Gateway which connects MQTT-SN nodes, such as constrained sensors, to the MQTT network. Figure 4 shows a basic MQTT architecture.

**CoAP** [53] (Constrained Application Protocol) is a generic web protocol designed for constrained environments with restricted network capacities and restricted devices, allowing these devices to communicate with the Internet or other constrained devices. It implements a compressed subset of the REST model implementing GET, POST, PUT, and DELETE operations over UDP.

CoAP reduces the message header and restricts message exchange, reducing the network overhead. It is very useful for Machine-to-Machine (M2M) communication, and for the Internet of Things (IoT).

CoAP can be easily translated to HTTP for seamless integration with existing Web systems, while reducing network requirements.

**Figure 5:**
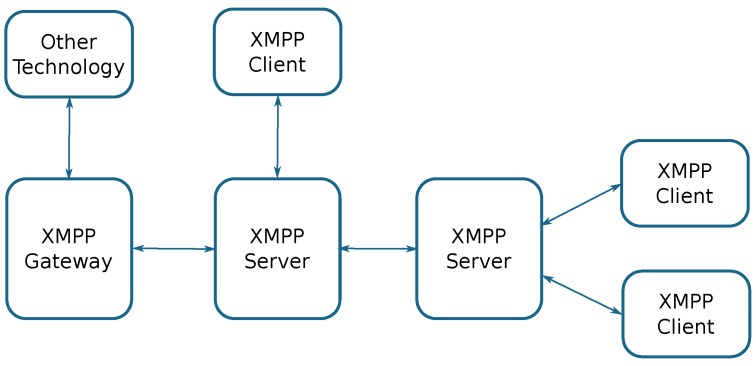
Basic XMPP (eXtensible Messaging and Presence Protocol) architecture.

**XMPP** [54] (Extensible Messaging and Presence Protocol) is a message-oriented communications protocol based on XML. Initially, it was called Jabber, and it was designed for instant messaging (IM). It allows federations among various XMPP servers, and even communication with different technologies using XMPP gateways. Currently, it is also used for VoIP, video, gaming, or even for IoT applications. Figure 5 shows the basic architecture of an XMPP-based system.

The specification for IoT is XEP-0323: Internet of Things—Sensor Data [55], which provides the architecture, basic operations, and data structures for sensor data communication, including a hardware abstraction model for the interconnection of constrained devices.

**sMAP** [56] (Simple Measuring and Actuation Profile) is an example of how RESTful web services can be simplified, while still allowing instruments and other producers of physical information to directly publish their data in a central server.

### 3.4. Message Format Layer

The Message Format layer presents all data encoding types to store and transmit structured data for IoT applications.

**XML** [57] (eXtensible Markup Language) is a markup language for encoding documents in a text format that is understandable by both human and machines. XML is designed to store data units called entities, where all data structures and document descriptions are achieved through markups. Using these markups, it is possible to create any logical data structure in an easy way.

**JSON** [58] (JavaScript Object Notation) is a format notation to encode structured data (attribute-value pair or array) using human-readable text. It was designed to replace XML by reducing its complexity. It is very common in web systems, especially in AJAX-style ones. It uses pairs (object_id:object_value) and brackets to provide complex object structuring for fitting data in a text document.

**EXI** [59] (Efficient XML Interchange) is a binary and compact representation of XML or JSON documents. It aims at resource-constrained devices and networks, attempting to reduce the size of the data and the computational requirements when compared to other compressors such as gzip. The EXI coder is based on events, and it follows a simplified Huffman coding to create a binary document.

**MessagePack** [60] is a binary serialization format that encodes messages faster and in a more compact manner than traditional methods such as JSON or XML. This is possible since small integer values are encoded in a single byte, while strings require only an extra byte to identify them. This simplifies the encoding process, but it has some limitations, such as the size of strings or numbers, the number of the key/value association map, etc.

Table 1 shows a representation of sensor data using XML and JSON encoding, and Table 2 shows a representation of sensor data using EXI and MessagePack encoding, respectively. We can observe that the EXI encoding and the MessagePack are much smaller (163 and 186 bytes) than the encoding achieved using XML (474 bytes) or JSON (357 bytes).

### 3.5. Semantic Layer

The Semantic layer presents all approaches that describe a logical representation of things in the IoT context. Therefore, in sensing approaches, we can find several representative examples:

**SensorML** [61] is an standard model based on XML encoding for describing sensors and measurement processes. It is developed by the Open Geospatial Consortium, describing a wide range of sensors for different types of architectures, including remote sensors, in-situ sensors and dynamic sensors, among others.

**Semantic Sensor Net Ontology** [62] describes sensors and observations, avoiding to describe domain concepts, location, time, etc. It is developed by the W3C Semantic Sensor Networks Incubator Group (SSN-XG).

**Web of Things** [63] specifies a data model to describe physical devices connected to the Web (Internet) using JSON encoding. It was created for the Mozilla project, and was formally submitted to W3C for discussion.

Taking into account that the previously described protocols, a sensor device must be able to cope with a subset of them to allow the exchange of data between the sensors and a central server.

## 4. Mobile Sensing Requirements

Normally, the sensing process is made through a Wireless Sensor Network (WSN) [64,65], which is composed by a set of nodes or sensors that collect data and send it towards a central sink or gateway. The latter carries out processing tasks, or merely resends collected data to a server for storage and processing. Usually, all sensors are resource-constrained devices, and the central sink or gateway has fewer restrictions, often being connected to the power network. Currently, most Wireless Sensor Networks are based on the IoT architecture since it allows us to work with constrained devices and restricted networks.

**Figure 6:**
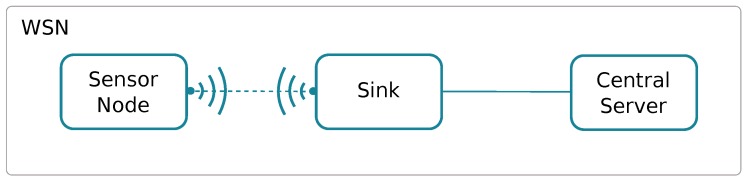
Wireless Sensor Network structure.

Figure 6 shows the basic structure of a Wireless Sensor Network. We can see that the communications link between the sensors and the sink/gateway is wireless, typically relying on ZigBee, and that the communications link between the sink/gateway and the central server is typically a more robust link, either wired (e.g., Ethernet) or wireless (e.g., Wi-Fi or Cellular).

**Figure 7:**
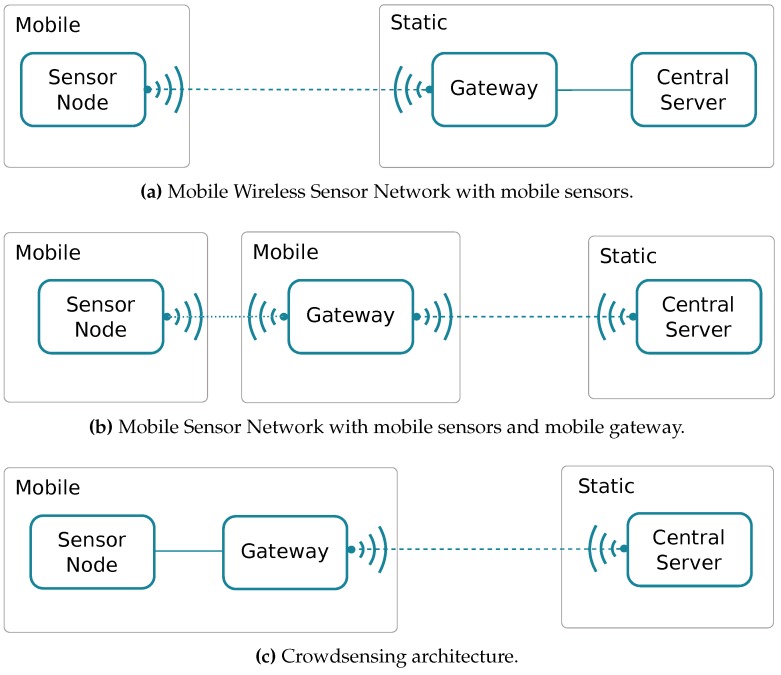
Different types of Mobile Sensor Networks’ structures.

**Figure 8:**
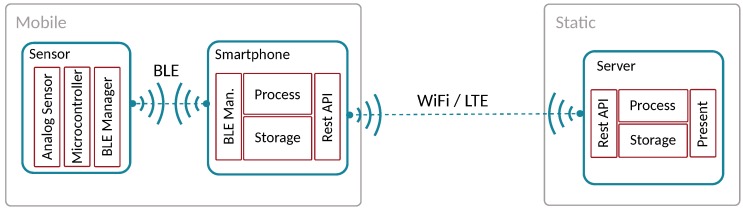
Crowdsensing architecture overview.

The support for mobility in a Wireless Sensor Network [66,67] can be achieved through different strategies, including sensor mobility, as shown in Figure 7a, or by having mobility on both sensors and gateway, as shown the Figure 7b. Finally, we have crowdsensing architectures where the gateway and the sensor are the same, or are packed together. Commonly, the best way to implement crowdsensing is through smartphones, since nearly all people carry one with them nowadays, and they are endowed with several sensors and communication interfaces. Figure 8 shows an example of a crowdsensing architecture where a smartphone is used as the gateway between the sensor and a Central Server.

Crowdsensing solutions need to be widely disseminated and adopted by users to be successful. In addition, to achieve such widespread acceptance, the impact on the users’ everyday activities must be low. This means that any deployed application must operate in the background and avoid consuming excessive resources, while requiring only a minimal user participation. Concerning the sensor itself, if external to the smartphone, it should be cheap, small, easy to use, and comfortable to carry.

Crowdsensing approaches have two basic architectural components [68]: a mobile component for the data acquisition process, and a central server for data storage and processing.

The mobile component must be able to collect environmental parameters, transmitting them towards the central server. The data acquisition process is based on smartphone sensors, or on small external sensors accessible via smartphone, and the transmission process usually relies on smartphone connectivity towards the Internet. Despite delegating transmission tasks on smartphones, external sensing devices must still be endowed with communication capabilities to transfer the collected data to the smartphone. Thus, the sensing device should be equipped with a wireless communications interface, being technologies such as Wi-Fi, Bluetooth, RFID, NFC, and ZigBee ZigBee is ok good candidate solutions.

The central processing server must be able to receive the transmitted data from the sensors, store and process the data, as well as properly present the obtained results to system managers. In addition, in some cases, they perform remote communication with the mobile devices for configuration tasks, thereby allowing to dynamically change the sensing behaviour.

**Figure 9:**
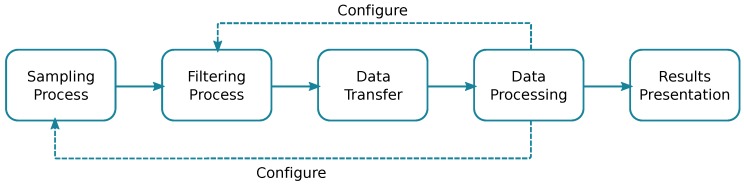
Crowdsensing steps.

Taking the aforementioned considerations into account, Figure 8 shows a basic hardware architecture applicable to air quality sensing applications that should include: (i) a mobile sensor; (ii) a smartphone; and (iii) a central server. The proposed architecture resembles various approaches from different authors [19,20,21,68]. Moreover, as shown in Figure 9, the crowdsensing process basically includes five different tasks: (i) sampling process; (ii) filtering process; (iii) data transfer; (iv) data processing; and (v) results presentation. Notice that all these tasks could be done by different hardware components; for instance, the filtering task could be done by the sensor, the smartphone, or even the central server, depending on the system characteristics. Moreover, characteristics associated to sensing, filtering, and transmission tasks could be defined based on parameters obtained from the processing step.

**Sampling process** refers to the process of capturing pollutant measurements, including the calibration process, where electrical signals are translated to pollution units, filters, fault detection and diagnosis, etc.

Sensor calibration, in Commercial Off-The-Shelf (COTS) sensors such as electrochemical ones, is a process that depends on the physical sensor characteristics, temperature, etc. Basically, electrical outputs must be translated to pollution units, and often there is no lineal relation. The calibration is commonly made in advanced laboratories, taking into account samples taken with different pollution levels and for different temperatures and humidity conditions [69,70]. However, in urban scenarios, the auto calibration procedure is too complicated because all sensors are distributed among different users. Alvear et al. [71] proposed a method to calibrate off-the-shell sensors using mathematical regressions based on high-accuracy samples obtained through the fixed stations deployed in a city. Once a mobile sensor is near to these stations, these samples are used to adjust the translation equation (electrical signal to pollution values).

Using COTS, the sampling error and its diagnostics can be a problem [71]. Nevertheless, when focusing on a Crowdsensing solution using a large number of mobile sensors (smart city scenario), this problem could be solved by accounting for redundant data and statistical analysis (i.e., Kriging allows us to deal with sampling error).

**Filtering process** refers to deleting redundant and/or wrong measurements caused by the sensors reading oscillations. By using mobile sensors, the filtering process also has to deal with temporal variations, as describe in [68,71], adjusting samples to a same temporal fragment.

**Data transfer process** refers to the upload of data from the sensor to the cloud (Central servers), including sensor-smartphone and smartphone-server communications. In is achieved through the previously described IoT protocols.

**Data processing** refers to the interpolation technique used to recreate a pollution distribution map. It could be made by different methods (Kriging, IDW, and Nearest neighbour Spatial Averaging) as described in [72]. Currently, the most used method is the Kriging interpolation technique, where a semivariogram is calculated for create a complete pollution map.

**Results presentation** refers to the way results are presented to the system administrator. The most useful representation is a graphical map for the target region.

**Figure 10:**
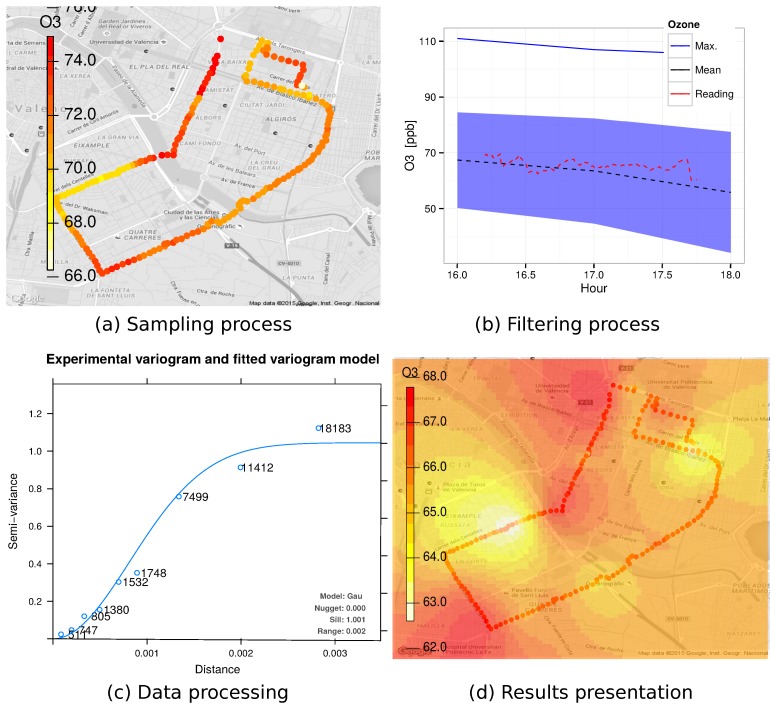
Data handling process as described in [68]: (**a**) sampling process; (**b**) filtering process after adjusting the temporal variations; (**c**) data analysis using a semivariogram of the captured data used for interpolating the entire area using the Kriging technique; and (**d**) pollution distribution map.

Figure 10 presents the data handling process, as the authors described in [68], showing the four processes for handling pollution information in order to recreate a complete pollution map for a certain target area.

### 4.1. Basic Smartphone Software Architecture

Concerning smartphones, they are devices widely used nowadays for any task, and characterised by powerful computing capabilities, large amounts of memory, and several embedded sensors and communication interfaces [73]. We consider smartphones as the best gateway option for connecting mobile sensors with a central server. In addition, they can perform CPU-intensive tasks such as data filtering or data fusion, simplifying sensor requirements and design to mere data acquisition and data relaying towards the smartphone.

**Figure 11:**
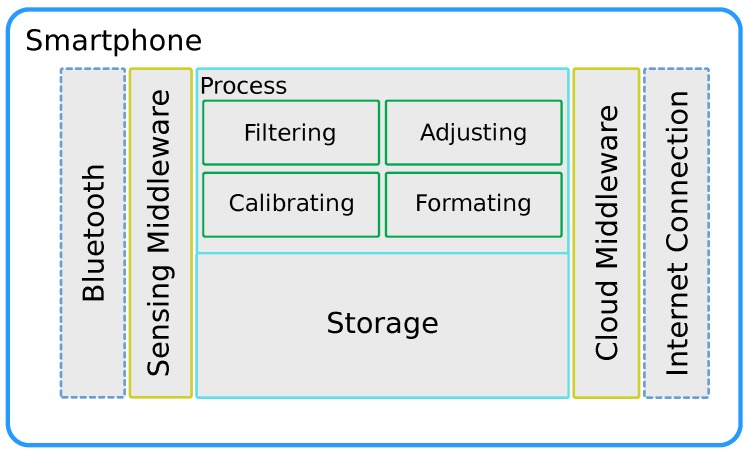
Smartphone software architecture overview.

Since the smartphone must act as a gateway between sensor and cloud server, it must manage at least two network interfaces: one to collect data from the sensor (Sensing middleware), and another one to upload data to a central server (Cloud middleware). Although both tasks must run independently, the data uploading process is often not made in real time, contrarily to the sensor data collection process, which is a task that should be done very frequently, especially if we aim at a simplified sensor design, as shown in Figure 11. Moreover, modules to process and store the collected data are also needed. By assuming that the smartphone becomes responsible for all the computationally intensive tasks, it becomes necessary to analyse which are the actual basic requirements when designing a mobile sensor for crowdsensing solutions.

### 4.2. Functional Requirements of the Mobile Sensor

Despite relying on smartphones for providing system intelligence, basic mobile sensor requirements still involve:

**Processing**: Bold is necessary The sensor must be able to process the measured data, perform basic filtering tasks, and transfer data to an external device. Anyway, in terms of processing power, requirements are low.

**Storage**: By assuming that a links towards a smartphone or a similar device is available, the sensor does not need to actually store large amounts of collected data. In fact, since data can be seamlessly relayed to the smartphone in real time, the sensor can limit its internal storage to only a few samples.

**Communication**: Sensors do not need to have a direct connection to the cloud server via Internet, but they still need to transfer the collected data to the smartphone. Thus, sensors require a communications link compatible with current smartphone technologies like Wi-Fi, Bluetooth, or NFC.

**Autonomy**: Sensors must be able to operate for long periods using a small power supply. Thus, energy optimisation becomes a key requirement to take advantage of small batteries.

**Size**: Sensors need to be transported by users, or to be quickly installed in vehicles (e.g., bicycle, motorcycles, and cars). Thus, they must be small enough for the sake of aesthetics and comfort.

**Price**: To be attractive to users, sensors must be as cheap as possible. Otherwise, it becomes difficult to meet the broad dissemination requirements of crowdsensing approaches.

### 4.3. Basic Mobile Sensor Design

To fulfil the technical requirements, a basic mobile sensor should be composed of a sensor device able to monitor the differences between different pollutant levels, a communications module for transferring the data collected, and a microcontroller/microcomputer acting as a central element for managing all tasks.

**Figure 12:**
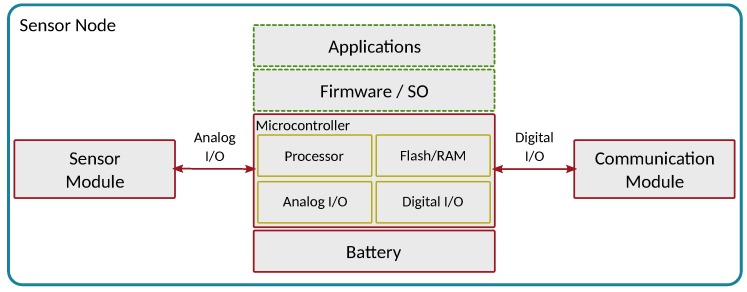
Mobile sensor design.

Figure 12 shows a basic mobile sensor design, and the main characteristics to consider. As shown, the sensor hardware module must be able to connect to a microcontroller/microcomputer, a connection that typically relies on an analog/digital port. Similarly, the communications module must also be connected to the microcontroller via an UART or USB port. Thus, the microcontroller becomes a central element in the sensing module, being responsible for managing the interactions between all the elements.

Overall, the sensing module must be equipped with different analog ports, UART/USB ports, a processor, and flash memory (ROM or RAM).

## 5. Overview of Available Hardware and Software

In recent years, the appearance of different embedded prototyping platforms, such as Raspberry Pi or Arduino, which are complemented by a large variety of compatible electronic components, paved the way for the creation of diverse applications related to IoT. When specifically focusing on environmental monitoring requirements, we find that there are different development options, including different types of sensor, and various communication interfaces.

Commercially, several companies offer small and yet powerful boards, along with a large variety of electronic components for personalising them according to user needs. In addition, there are various companies offering extra modules or add-ons such as Seeedstudio [74], which offers their own sensors for developing personalised frameworks based on Grove technologies. Similarly, Adafruit [75] provides different embedded platforms, as well as all kinds of electronic components, including personalised add-ons for batteries, communication modules, and sensor boards compatible with the most widely extended platforms: Raspberry Pi, Arduino, BeagleBone, Intel Edison, or Intel Galileo.

Focusing on final solutions, TST [76] offers products in the field of Smart Cities (Waste Management, Industrial Control, Light Control, etc.), basing their solutions on their own hardware platform. Likewise, Libelium [23] is a company providing various products in the field of monitoring (Environment monitoring, Agriculture, Water monitoring, etc.); most of the components, and the programming tool used, are based on the Arduino platform. However, from a crowdsensing perspective, the solutions offered are inadequate due to the relatively large sizes of the devices, being mostly oriented for public infrastructure deployment.

Based on the state of the art, we now provide an in-depth analysis of the different hardware and software components applicable to our mobile air quality sensing context.

To enable air quality data acquisition, specialised pollution sensors must be connected to a microcontroller or microcomputer via an analog or digital port. Moreover, for communication tasks, the microcontroller/microcomputer must be connected to the communications module via an USB port or UART interface. In this sense, available options will depend on the microcontroller/ microcomputer characteristics.

### 5.1. Microcontroller/Microcomputer-Based Embedded Systems

Despite the lightweight processing constraints, there are several options available for embedded systems acting as central elements in the sensor design. In fact, it is possible to use microcomputers such as Raspberry Pi, BeagleBone, or Intel Edison, which use a standard operating system, and that allow developing applications for sensing tasks in a straightforward manner. Alternatively, it is possible to use a microcontroller board such as Arduino, and develop application-specific firmware instead.

**Raspberry Pi** is one of the most popular microcomputers worldwide. It is a low-cost and small-sized computer that allows connecting standard PC peripherals including a monitor, a keyboard, and a mouse. It was designed to explore computing, and it supports different Operating Systems: Raspbian, which is based on Debian, and also Ubuntu Mate or Windows 10 IoT Core, thereby allowing to use several programming languages. In addition, all Raspberry Pi versions benefit from several input/output ports operating at 5 Volts, thus being ideal for all sorts of IoT projects.

**Figure 13:**
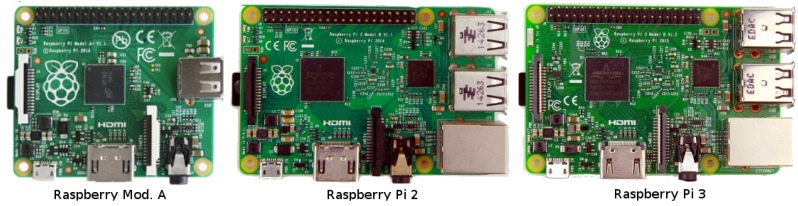
Overview of different Raspberry Pi models.

There are different versions of the Raspberry Pi, as shown Figure 13, being model A and type 2 the most commonly used. They have different characteristics, e.g., Type B offers better performance in terms of memory and processing, but Model A consumes much less power. Recently, Raspberry Pi 3 has appeared, offering better features than previous versions, being the major difference the integration of a Bluetooth and a Wi-Fi module, thereby facilitating communication tasks in the scope of IoT projects.

**BeagleBone** is a small computer running a Linux Operating System called Angstrom, and supporting various software distributions such as Android or Ubuntu. It has an USB port for connecting distinct peripherals, along with an HDMI port for video connection, allowing to use it as a regular computer. It has two 46 pin headers which operate at 3.3 V, allowing to augment the available functionalities by connecting different digital or analog devices like sensors or actuators.

**Figure 14:**
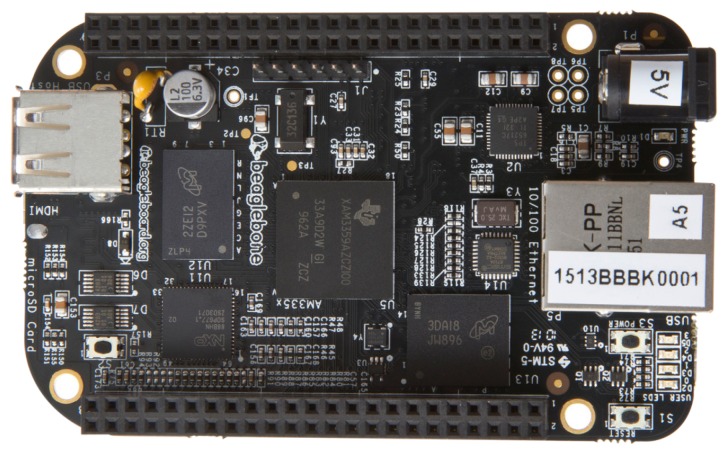
Beaglebone Black microcomputer overview.

Commercially, we can find several Beaglebone versions, being Beaglebone Black the most commonly used (see Figure 14).

**Figure 15:**
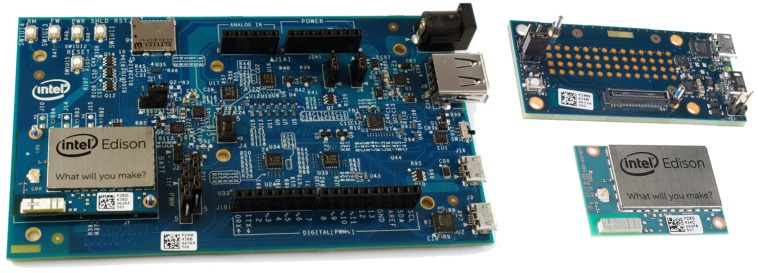
Intel Edison with two extension boards.

**Intel Edison** is a tiny but powerful computer developed by Intel. It is designed for IoT applications, targeting at both prototypes and commercial solutions with performance constraints. It supports a modified Linux distribution (Yocto) as its Operating System, and it integrates both Wi-Fi and Bluetooth 4.0 interfaces. In addition, it can benefit from two types of expansion board: an Arduino Expansion board, and a mini breakout board (see Figure 15).

**Figure 16:**
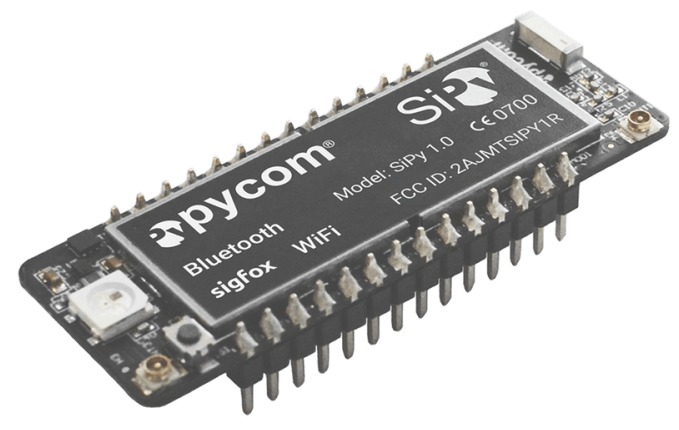
Pycom module.

**Pycom** [77] is a microcontroller based on the ESP32 chip with 24 GPIO pins, 2 UARTs, 1 SPI and 1 I2C port, using a firmware based on micropython. It can be equipped with several communication interfaces such as Wi-Fi, Bluetooth Low Energy, LoRA, and Sigfox. Moreover, using an expansion board, it can integrate an SD Card, as well as different sensors such as Gyroscope, Accelerometer, or GPS (see Figure 16).

**Arduino Uno** is an open source prototyping platform characterised by easy-to-use hardware and software. It has several analog and digital input/output pins to connect sensors, actuators or complementary boards, allowing to create a wide variety of IoT solutions. Arduino has its own programming language based on Wiring, and its own Arduino Software based on Processing [78]. It has a central microcontroller, and an USB port for programming and to supply power.

**Figure 17:**
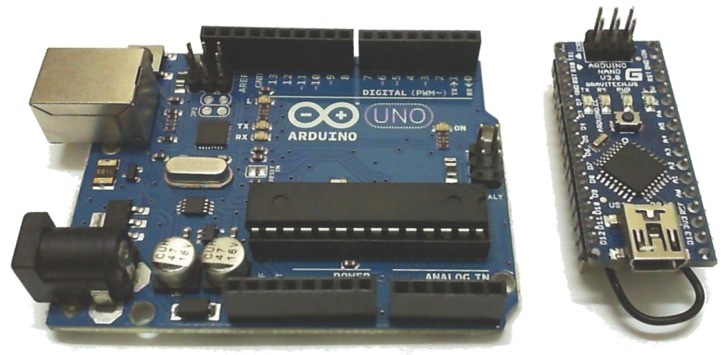
Arduino Nano module (**right**) and Arduino Uno module (**left**).

**Arduino nano** is a tiny prototyping platform that maintains the Arduino Uno concept, using the same programming languages and the same libraries. It was also developed for prototyping solutions, but it is smaller than the standard Arduino, and it has less available memory (see Figure 17).

Table 3 allows comparing these five embedded systems by providing a summary of the most significant technical details.

#### Operating Systems for IoT Microcomputers

The choice of an adequate Operating System is a very important issue in the design of a sensor since it will shape the corresponding software architecture. Thus, we now proceed by analysing the different operating systems available for the microcomputers referred above.

**Raspbian** is the most common operating system designed for Raspberry Pi. It is supported by all Raspberry Pi versions, and it has two versions: (i) a complete version with a graphical interface and many development tools which facilitate the development of solutions, but that consumes a lot of resources; and (ii) a lite version, without graphical interface, and with just a basic set of preinstalled software, allowing to add only those packages that are actually required.

Since it is a Linux-based operating system, it supports several programming languages like C, C++, Java, Scratch, Python, or BASH.

**Ubuntu MATE** is an option for Raspberry Pi microcomputers that is supported by model 2 and model 3. This operating system attempts to be simple from the end user perspective, integrating several entertainment applications, although it is also possible to add development tools to it.

**Angstrom** is a modified Linux optimised for Beaglebone microcomputers. It has no graphical interface, and it supports several programming languages such as Python, C, Java, or BASH. In addition, it has its own programming language called BoneScript, which is based on the node.js language.

**Yocto Project** is a complete embedded Linux development environment with tools and methods to facilitate the creation of embedded systems. It can be configured to run Arduino-based or Linux-based programs, thereby offering a great flexibility.

**Micropython** [79] is a Python 3.5 implementation optimised for running in microcontrollers. It allows interacting via a prompt, executing commands or running scripts in an autonomous way. It is entirely compatible with python, and it includes modules for accessing low-level hardware.

**Windows 10 IoT Core** is a Windows 10 based operating system oriented to Internet of Things projects using small devices. It is supported by Raspberry Pi 2 and 3, Arrow DragonBoard 410c, and MinnowBoard MAX. Windows 10 IoT Core relies on the rich, extensible Universal Windows Platform (UWP) API for building solutions. It can be used together with the Visual Studio environment for programming.

Table 4 shows a brief comparison of the different Operating Systems currently available.

### 5.2. Air Pollution Sensors

**Figure 18:**
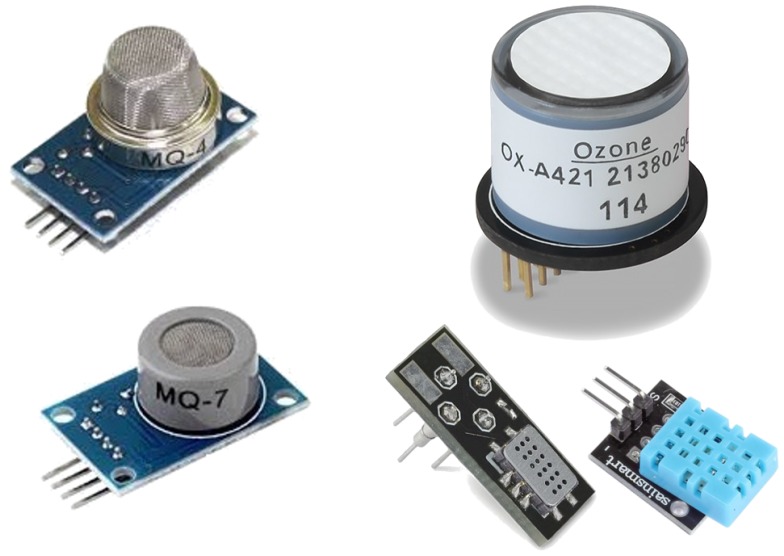
Different types of air pollution sensors.

Nowadays, we can find a wide variety of sensing technologies (see Figure 18) for gas detection (Metal oxide semiconductor, polymer, carbon nanotubes, moisture absorbing materials, Optics, Acoustics, etc.) as shown in [80]. Each technology has different properties, calibration processes and costs, among other characteristics, and so a comparison between these different technologies is required.

To evaluate the different sensing technologies, we must mainly consider some characteristics, especially when focusing on the design of small mobile sensors: (i) sensibility, which refers to the range of values that the sensor can measure; (ii) selectivity, which is the capability of reacting only to the target gas; (iii) linearity, which is the rate of change with respect to gas variations; (iv) response time, which is the time required to start measuring correctly; (v) power consumption; and (vi) price.

In the market, we can easily find various gas sensors for air pollution monitoring, being the three following types of sensors the most common: electrochemical, semiconductor, and infrared. These have a wide range of prices and characteristics. Below we provide more details about each of these sensor types:

**Electrochemical gas sensors** measure the concentration of some air pollutant by oxidizing or reducing its internal porous membrane, thereby producing current changes. Usually these sensors behave quite linearly, allowing to make accurate measurements. They typically operate at 5 V, having a power consumption of about 600 mW; most pollution sensors in this category cost between $100 and $400.

**Semiconductor gas sensors** are the most common gas sensors because of their low cost and high sensitivity. They have an internal conductive material that increases their conductivity level in the presence of a specific air pollutant. These sensors are nonlinear and have a low selectivity, adding difficulty to the monitoring process. Usually they operate at 5 V, having a power consumption in the 500–900 mW range, and they cost between $10 and $35.

**Moisture absorbing material sensors** are used for measuring temperature and humidity. Their dielectric constant varies according to the water content in the environment. They operate at 5 V, having a power consumption of about 0.5 W. In addition, they are very cheap, with a price of about $5.

**Infrared sensors** measure gas/element variations by detecting interferences in an infrared laser. They are specially adequate for monitoring pollutants such as fine particulate matter sized less than 10 micrometers (PM${}_{10}$), or fine particulate matter sized less than 2.5 micrometers (PM${}_{2.5}$). They usually operate at 5 V, having a power consumption of about 1W, and their cost is about $40.

Table 5 provides a brief summary of the most significant aspects of these different types of sensors for comparison purposes.

### 5.3. Communications Modules

**Figure 19:**
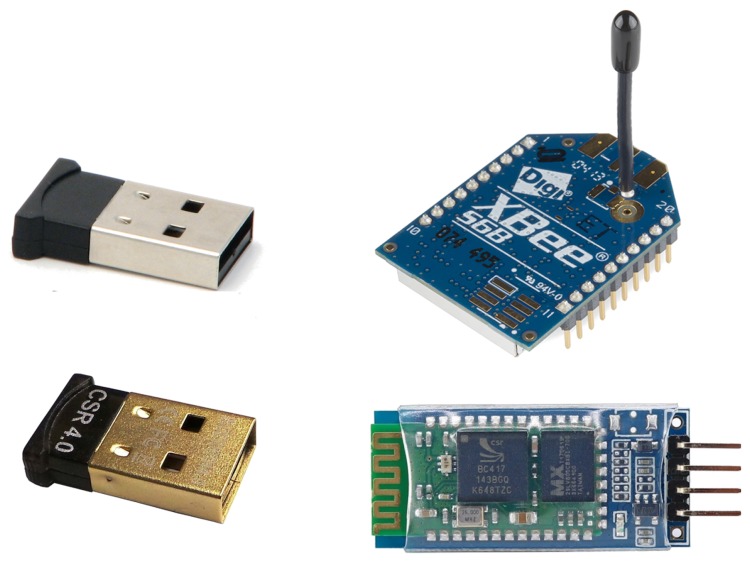
Example of some communication modules.

Although the RFID [81] standard was developed for IoT solutions, there are currently several options available for providing communications between the sensors and the mobile terminal (usually a smartphone), as shown in Section 3. Figure 19 shows some communication modules examples. We now proceed to analyse the technical characteristics associated to the different available options.

The main characteristics to consider are: (i) distance, that is, the wireless coverage range; (ii) communication type, which refers to the characteristics of the channel over which messages are transmitted—usually two types are considered, i.e. serial-based, when a communications channel is open to transmit a stream of data, or message-based, when the data are transmitted via a unique message; (iii) message size, which refers to the maximum size of the message when the communication is message-based; (iv) power consumption; and (v) price.

Table 6 shows a brief summary of the most significant aspects to consider in terms of communication modules.

We can observe that all options are relatively cheap, with prices in the range from $40–$50, but the more widely used in the market, i.e., Wi-Fi and both Bluetooth and Bluetooth Low Energy, are the cheapest ones, with prices in the range $10–$15. In terms of power consumption, the best option is Bluetooth Low Energy (0.05 W), although ZigBee, LoRA and NFC also exhibit low power consumption levels. In terms of bitrate, the best performing technology is Wi-Fi, being that typical wireless sensing technologies, such as LoRA, ZigBee, or SIGFOX, have a low bitrate. Regarding the communications type, there are two options: (i) Wi-Fi and Bluetooth, which open a serial communications channel for transmitting a stream of bytes; and (ii) Bluetooth Low Energy, ZigBee, Sigfox, LoRA, and NFC, which transmit a message per iteration.

## 6. Mobile Air Pollution Sensor Design

The design of a small and cheap mobile sensor is a basic requirement for air pollution monitoring. After analysing the main technical characteristics of hardware components available in the market, it quickly becomes evident that there are several options for creating a mobile air quality sensor for crowdsensing in the Internet of Things context.

If focusing on the mobile sensor/smartphone wireless connection, the best option is using Bluetooth Low Energy since it is able to fulfil all requirements (low power consumption, low price, and small-sized modules) ’(’ could be better whereas all other options have different drawbacks. For instance, the ZigBee technology, despite being the most extended technology for wireless sensor networks due to its low power consumption, is not supported by current smartphones. The SIGFOX technology has very strict restrictions regarding the number of messages that it is possible to generate per time slot, thus having little applicability to our aims. The Wi-Fi technology, although being widely used and having a large coverage range, consumes more power and is typically used for Internet connectivity. Finally, the major problem of the NFC technology is its coverage range (only about 4 cm).

In terms of sensor device prototyping, the best option for creating a small and cheap mobile sensor for air pollution monitoring is the Semiconductor gas option. Notice that it is cheaper than the electrochemical sensor, and even though the latter is more accurate, the error introduced can be mitigated by combining information from other nearby users. Concerning infrared sensors, they are only applicable to a specific type of pollutant (fine particulate), while moisture absorbing sensors are mostly used to measure temperature and humidity.

Next, we propose and evaluate some possible configurations for the different studied boards using a semiconductor gas sensor for pollution monitoring, and a Bluetooth Low Energy interface as the transmission technology. For all the proposed hardware configurations, it is possible to use an external USB charger as a power supply to offer more autonomy, while maintaining the support for mobility.

**Figure 20:**
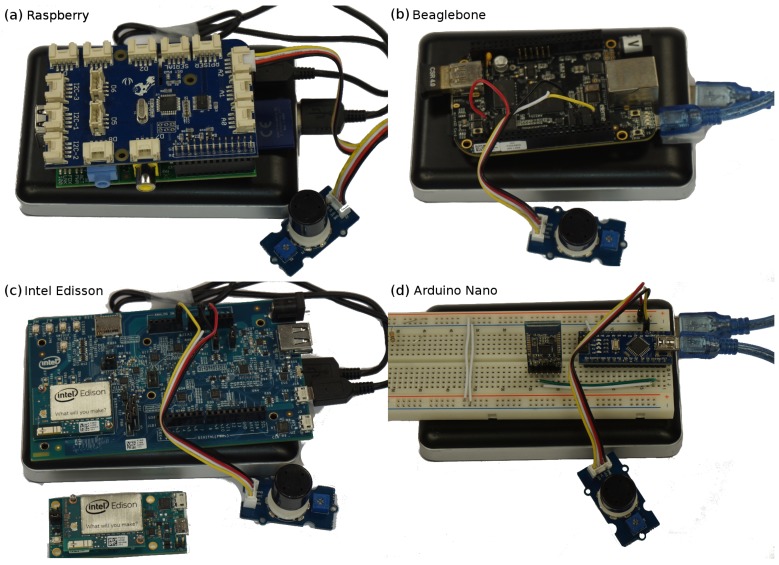
Proposed mobile sensing solutions for air quality monitoring.

Figure 20 shows the four solutions we have developed to evaluate the different hardware options, and Table 7 shows a comparison between these solutions.

The first hardware option we propose is based on the Raspberry Pi platform (see Figure 20a). Since it has no analog ports, it has to be provided with an analog/digital converter. For this purpose, we propose using GrovePi [82], which is an extension board that allows connecting several analog/digital grove ports to a Raspberry Pi in an easy way. Furthermore, since the Raspberry Pi has several USB ports, we propose using a standard USB Bluetooth module for Raspberry Pi 2, or the built-in Bluetooth module for Raspberry Pi 3. With this solution, it becomes possible to run several programming languages, as it is possible to install a Linux or a Windows 10 IoT operating system, and there are a lot of development efforts around it. This configuration has a power consumption of about 2000 mW, a total weight of 200 g, and it costs about $90. Overall, it is the most power-hungry solution among the four proposed, but it becomes the best option for quick prototyping due to its flexibility and large community of developers.

The second hardware approach relies on the BeagleBone board (see Figure 20b). It has several analog ports, allowing us to connect the sensor directly to this board without any intermediate device. In addition, since the BeagleBone has a USB port, it is possible to use a standard USB Bluetooth module. It runs a Linux-based operating system, allowing it to run different programming languages, but there are not too many developments or projects focusing on this solution. This configuration has a power consumption of about 1500 mW, a total weight of 150 g, and it costs about $110, thus being one of the most expensive options. It is not a very useful prototyping platform since it has characteristics that are similar to the Raspberry Pi, but it has a smaller developer community and less support.

The third hardware solution we propose is based on the Intel Edison platform (see Figure 20c). It has an embedded Bluetooth interface, but it does not provide an analog port to directly connect the sensor. For this purpose, it is possible to use: (i) the Arduino expansion board; or (ii) the Breakout board [83]. The first sensor connection option is very simple, however the sensor becomes excessively large. Regarding the second option, the overall size remains small, but it is necessary to make an ad hoc circuit to connect the sensor. The Edison board supports a Linux-based operating system (Yocto) including the possibility to run Arduino-based scripts. For this last configuration, the power consumption is of about 1000 mW, the total weight is 200 g, and it costs about $130, making it the most expensive option. It is useful mostly for end solutions due to its price.

The last embedded solution we propose is based on the Arduino platform (see Figure 20d). Since it was designed for these types of solutions, it becomes easy to connect a sensor via the existing analog ports; nevertheless, USB ports are not available, and so a Bluetooth module must be connected via an UART port for both Arduino Uno and Arduino Nano boards. This solution only runs Arduino-based scripts, reducing the programming flexibility, but we can find a lot of developments using this platform.

For the Arduino Uno solution, the power consumption is about 600 mW, the total weight is 150 g, and it costs about $55. This option is improved by the Arduino Nano solution, which has a power consumption of about 600 mW, a total weight of 60 g, and it costs about $50. The latter option is better than all others in terms of consumption, weight, and price, having as its only drawback the limited memory/CPU resources. It is also useful for restricted environments where the power consumption is very limited.

## 7. Conclusions and Future Work

Smart Cities is a trending topic, and many research efforts are being made worldwide to progress towards that new paradigm which encompasses several areas including smart government, smart transport, smart environment, and smart grid. Since air pollution is considered to be one of the most significant health risks worldwide, smart environment obviously becomes a very important area in the Smart City context.

Although air monitoring stations have been sparsely deployed in most large cities for controlling pollution levels, these are not enough to provide a detailed view of the pollutants’ distribution in a city. In this sense, crowdsensing emerges as a good option to monitor the different pollutants by combining small mobile sensors and smartphones. Since current smartphones have much memory and high computing capabilities, mobile sensors can be kept minimal, focusing on data acquisition tasks alone.

In this paper, we have analysed the most relevant Internet of Things architectures and protocols, along with the requirements of an embedded mobile sensor platform from a crowdsensing perspective, identifying the basic tasks the sensor must be able to perform. Besides, an analysis of the hardware architecture requirements has been done, and candidate off-the-shelf hardware components have been analysed. Finally, several complete hardware configurations meeting all the design requirements have been developed and compared in terms of power consumption, weight, and cost. Overall, we have found that the Arduino Nano platform, despite having very limited resources, is able to fulfil the established requirements, thus being the most recommendable alternative in terms of price, weight, and power consumption features.

Regarding other hardware alternatives, microcomputers such as Raspberry Pi, BeagleBone, or Intel Edison are more powerful and flexible by supporting a standard Operating System, thereby allowing to quickly deploy any application. We believe that the Raspberry Pi solution can be the best option for quick prototyping. For more professional solutions, requiring higher processing capacity, the Intel Edison becomes a better option, although imposing a higher overhead in terms of development time. Finally, Arduino becomes an option for very restricted environments.

Overall, we find that a hardware solution applicable to all IoT contexts, and meeting low size and low power requirements, along with adequate communication interfaces and battery capacity, is still missing, although in years to come many more products are expected.

The next steps in this research line are to test the adequacy of the proposed solutions not only in the mobile user context, but also in other contexts including coupling these sensors to vehicles such as cars, buses, bikes, or even flying vehicles (multicopters).

## Figures and Tables

**Table 1:** Example of data representation using typical encoding types.

XML Encoding (474 bytes)	JSON Encoding (357 bytes)
<?xml version="1.0" encoding="UTF-8" ?> <trace> <id>trace1</id> <values> <captures> <latitude>39.470577</latitude> <longitude>-0.3336604</longitude> <ozone>56</ozone> </captures> <captures> <latitude>39.470652</latitude> <longitude>-0.3343365</longitude> <ozone>68</ozone> </captures> <captures> <latitude>39.470892</latitude> <longitude>-0.3359987</longitude> <ozone>59</ozone> </captures> </values> </trace>	{ "trace": { "id": "trace1", "values": { "captures": [ { "latitude": "39.470577", "longitude": "-0.3336604", "ozone": "56" }, { "latitude": "39.470652", "longitude": "-0.3343365", "ozone": "68" }, { "latitude": "39.470892", "longitude": "-0.3359987", "ozone": "59" } ] } } }

**Table 2:** Example of data representation using compressed (binary) encoding types.

EXI Encoding (163 bytes)	MessagePack Encoding (186 bytes)
80 40 67 47 26 16 36 5a 80 24 06 d2 c9 50 08 84 3a 39 30 b1 b2 98 80 ee cc 2d 8e ac ae 75 00 48 25 8d 85 c1 d1 d5 c9 95 ce a0 00 00 96 c6 17 46 97 47 56 46 5a 80 44 2c cc e4 b8 d0 dc c0 d4 dc dc 0a 6c 6f 6e 67 69 74 75 64 65 a8 04 43 0b 4c 0b 8c cc cc cd 8d 8c 0d 00 66 f7 a6 f6 e6 5a 80 44 10 d4 d9 00 0c 02 cc ce 4b 8d 0d cc 0d 8d 4c 80 0c 83 0b 4c 0b 8c cc cd 0c cc cd 8d 40 0d 01 0d 8e 10 00 c0 2c cc e4 b8 d0 dc c0 e0 e4 c8 00 c8 30 b4 c0 b8 cc cc d4 e4 e4 e0 dc 00 d0 10 d4 e5 ea 80	81 a5 74 72 61 63 65 82 a2 69 64 a6 74 72 61 63 65 31 a6 76 61 6c 75 65 73 81 a8 63 61 70 74 75 72 65 73 93 83 a8 6c 61 74 69 74 75 64 65 a9 33 39 2e 34 37 30 35 37 37 a9 6c 6f 6e 67 69 74 75 64 65 aa 2d 30 2e 33 33 33 36 36 30 34 a5 6f 7a 6f 6e 65 a2 35 36 83 a8 6c 61 74 69 74 75 64 65 a9 33 39 2e 34 37 30 36 35 32 a9 6c 6f 6e 67 69 74 75 64 65 aa 2d 30 2e 33 33 34 33 33 36 35 a5 6f 7a 6f 6e 65 a2 36 38 83 a8 6c 61 74 69 74 75 64 65 a9 33 39 2e 34 37 30 38 39 32 a9 6c 6f 6e 67 69 74 75 64 65 aa 2d 30 2e 33 33 35 39 39 38 37 a5 6f 7a 6f 6e 65 a2 35 39

**Table 3:** Comparison of different processing module components.

Board	CPU Speed	Memory/Storage	Power Comp.	Ports A/D	Size and Weight	USB Ports/Wireless Interfaces	Price	Operating Systems
Raspberry Pi Model A	700 MHz	256 MB/SD (4 GB)	0.8 W	0/30	6.5 × 5.5 cm/100 g	1 USB port/-	$25	Raspbian
Raspberry Pi 2	900 MHz	1 GB/SD (4 GB)	1.5 W	0/30	8.4 × 5.5 cm/136 g	4 USB ports/-	$35	Raspbian Ubuntu Mate Windows 10 IoT
Raspberry Pi 3	1.2 GHz	1 GB/SD (4 GB)	1.8 W	0/30	8.4 × 5.5 cm/136 g	4 USB ports/Wi-Fi and Bluetooth	$40	Raspbian Ubuntu Mate Windows 10 IoT
Beagle Bone	720 MHz	512 MB/SD (4 GB)	1.0 W	14/6	8.4 × 5.5 cm/100 g	1 USB port/-	$75	BeagleBone Linux
Intel Edison	500 MHz	1 GB/SD (4 GB)	0.6 W	14/6	5.9 × 2.8 cm/82 g	-/Wi-Fi and Bluetooth	$90	Yocto Project
Pycom	32 MHz	1 kB/32 kB	0.2 W	14/6	6.8 × 5.4 cm/100 g	Wi-Fi, Bluetooth and LoRa	$45	Micropython
Arduino Uno	32 MHz	1 kB/32 kB	0.2 W	14/6	6.8 × 5.4 cm/100 g	-/-	$25	Processing-based
Arduino Nano	16 MHz	512 B/16 kB	0.2 W	14/6	4.2 × 1.8 cm/20 g	-/-	$10	Processing-based

**Table 4:** Comparison of different operating systems.

Operating System	Booting Time (s)	Min. Memory (MB)	Graphical Interface	Type	Programming Languages
Raspbian	25	150	Yes	Multi-thread	C Java Python Scratch
Raspbian Lite	15	50	No	Multi-thread	C Java Python Scratch
Ubuntu MATE	30	200	Yes	Multi-thread	C Java Python
Yocto Project	20	150	No	Multi-thread	C Java Python Arduino-based
Angstrom Linux	15	100	No	Multi-thread	C Java Python Bonescript
Windows 10 IoT	35	250	No	Multi-thread	Visual Studio C
Micropython	<1	<1	No	Multi-thread	Python -
Processing-based	<1	<1	No	Single-thread	Wiring -

**Table 5:** Comparison of different sensing module components.

Sensor Type	Sensitivity	Selectivity	Linearity	Response Time	Power Comp.	Size (cm)	Price
Electrochemical	Medium	Medium	High	Medium	0.6 W	2.0 × 4.0	$200
Semiconductor	High	Low	Low	Low	0.5 W	2.0 × 4.0	$10–$35
Moisture Absorbing	High	Medium	High	High	0.5 W	2.0 × 4.0	$5
Infrared	High	Low	Medium	High	1.0 W	15.0 × 10.0	$40

**Table 6:** Comparison of different network module components.

Module	Communication Type	Max. Message Size	Data Rate	Distance	Power Comp.	Price
Wi-Fi	Serial-based	-	+54 Mbps	100 m	0.5 W	$10
NFC	Message-based	32 kB	424 Kbps	0.04 m	0.1 W	$35
ZigBee	Message-based	128 bits	250 Kbps	100 m	0.1 W	$40
SIGFOX	Message-based	96 bits	140 msg/day	+1 km	0.3 W	$60
LoRa	Message-based	0.1, 1 or 10% TimeOnAir	250–5400 bps	2–15 km	0.1 W	$45
Bluetooth	Serial-based	-	+2.1 Mbps	10 m	0.2 W	$10
Bluetooth LE	Message-based	160 bits	1 Mbps	10 m	0.05 W	$15

**Table 7:** Comparison of the four different solutions proposed.

Module	Extras	Network	Power Comp.	Weight	Price	Flexib.	Develop.	Comp. Power
RPi 3	+converter	–	2000 mW	200 g	€90	★★★	★★★	★★★
Beaglebone	–	+BLE usb	1500 mW	150 g	€110	★★★	★★★	★★★
Intel Edison	+breakout +expansion	–	1000 mW	100 g 200 g	€130	★★★	★★ ★	★★★
Arduino	+circuit	+BLE uart	600 mW	60 g	€55	★★★	★★★	★★★
